# Chronic hepatitis B and C infections in the Netherlands: estimated prevalence in risk groups and the general population

**DOI:** 10.1017/S0950268819000359

**Published:** 2019-03-14

**Authors:** J. Koopsen, J. E. van Steenbergen, J. H. Richardus, M. Prins, E. L. M. Op de Coul, E. A. Croes, J. Heil, F. R. Zuure, I. K. Veldhuijzen

**Affiliations:** 1 Center for Infectious Disease Control, National Institute for Public Health and the Environment (RIVM), Bilthoven, The Netherlands; 2 Department of Infectious Diseases, Research and Prevention, Public Health Service of Amsterdam, Amsterdam, The Netherlands; 3 Center for Infectious Diseases, Leiden University Medical Center, Leiden, The Netherlands; 4 Department of Public Health, Erasmus MC, University Medical Center Rotterdam, Rotterdam, The Netherlands; 5 Division of Infectious Disease Control, Municipal Public Health Service, Rotterdam-Rijnmond, The Netherlands; 6 Department of Internal Medicine, Amsterdam Infection and Immunity Institute (AI&II), Academic Medical Center (University of Amsterdam), Amsterdam, The Netherlands; 7 Netherlands Institute of Mental Health and Addiction (Trimbos Institute), Utrecht, The Netherlands; 8 Department of Sexual Health, Infectious Diseases and Environmental Health, Municipal Public Health Service South Limburg, Geleen, The Netherlands; 9 Department of Medical Microbiology, Care and Public Health Research Institute (CAPHRI), Maastricht University Medical Center (MUMC+), Maastricht, the Netherlands

**Keywords:** hepatitis B virus, hepatitis C virus, migrants, prevalence

## Abstract

Chronic hepatitis B virus (HBV) and hepatitis C virus (HCV) infections are usually asymptomatic for decades, thus targeted screening can prevent liver disease by timely diagnosis and linkage to care. More robust estimates of chronic HBV and HCV infections in the general population and risk groups are needed. Using a modified workbook method, the total number of ever chronically infected individuals in the Netherlands in 2016 was determined using population size and prevalence estimates from studies in the general and high-risk population. The estimated 2016 chronic HBV infection prevalence is 0.34% (low 0.22%, high 0.47%), corresponding to approximately 49 000 (low 31 000, high 66 000) HBV-infected individuals aged 15 years and older. The estimated ever-chronic HCV infection prevalence is 0.16% (low 0.06%, high 0.27%), corresponding to approximately 23 000 (low 8000, high 38 000) ever-chronic HCV-infected individuals. The prevalence of chronic HBV and HCV infections in the Netherlands is low. First-generation migrants account for most infections with 81% and 60% of chronic HBV and HCV infections, respectively. However, about one-fifth of HCV infections is found in the general population at low risk. This method can serve as an example for countries in need of more accurate prevalence estimates, to help the design and evaluation of prevention and control policies.

## Introduction

Worldwide, it is estimated that 248 million people are chronically infected with hepatitis B virus (HBV) [1] and that 71 million are chronically infected with hepatitis C virus (HCV) [2]. HBV and HCV infection prevalence varies across different regions. In the countries of the European Union (EU) and European Economic Area (EEA), approximately 4.7 million people live with chronic HBV and 5.6 million people are anti-HCV positive, which is 0.9% (95% CI 0.7–1.2) and 1.1% (95% CI 0.9–1.4) of the population, respectively [3]. In 2015, an estimated 702 000 and 495 000 people worldwide died of chronic hepatitis B or hepatitis C or related liver diseases, respectively [4]. In Europe (Global Burden of Disease region), this was 74 500 and 114 600 for HBV and HCV, respectively [4]. Globally, chronic infections with HBV and HCV are responsible for the majority of cases of liver cirrhosis (57%) and hepatocellular carcinoma (78%) [5]. Because chronic HBV and HCV infections are usually asymptomatic for several decades before irreversible damage is evident, they remain undetected until treatment options become limited. Approximately 20% of people with untreated chronic HBV and 15–30% of people with chronic HCV infection are estimated to develop cirrhosis within 20 years [6, 7]. It was estimated that in the Netherlands approximately 500 people died annually between 2002 and 2015 due to the consequences of chronic HBV or HCV infection [8]. This end-stage disease and mortality is to a large extent avoidable as safe and effective antiviral treatment became available for chronic active HBV infections and direct-acting antivirals (DAAs) are accessible for all patients with chronic HCV infection in the Netherlands in recent years.

To eliminate HBV and HCV as a public health threat the World Health Organization (WHO) set targets, including 90% diagnosis coverage of all individuals chronically infected with HBV and HCV by 2030 [9]. In 2016 the Health Council of the Netherlands advised targeted screening within high-risk populations [10]. Previous studies estimated the prevalence of chronic HBV infection, anti-HCV antibodies and chronic HCV infection in the Netherlands. A national serosurvey in 2006–2007 in the Netherlands estimated a weighted seroprevalence of 0.2% for HBsAg and of 0.3% for anti-HCV antibodies in the general Dutch population [11, 12]. However, because HBV and HCV infections are concentrated within high-risk groups such as migrants, men who have sex with men (MSM) and people who inject drugs (PWID) – which are often underrepresented in (voluntary) national serosurveys [13] – these estimations are possibly an underestimation. In the past 5 years, multiple prevalence estimates from studies among migrants in the Netherlands became available [14–19]. To design appropriately targeted screening interventions, robust prevalence estimates are needed for the general population and different risk groups. The European Centre for Disease Prevention and Control has called for more accurate prevalence estimates in EU/EEA countries to better inform prevention and control policies [20]. Therefore, we aim to determine the overall and risk group-specific prevalences of chronic HBV (HBsAg-positive) and HCV (ever HCV-RNA positive) in the Netherlands in 2016.

## Methods

### Workbook method

The prevalence of chronic HBV and HCV infection in the Netherlands in 2016 was estimated by using the workbook method. The workbook method has been developed by the Joint United Nations program on HIV/AIDS (UNAIDS) and the WHO, in collaboration with the UNAIDS Reference Group on Estimates, Modeling and Projections for estimating the HIV/AIDS prevalence in low endemic countries with concentrated epidemics [21]. The following risk groups and a group at low risk were distinguished:

1. First-generation migrants originating from low-intermediate to high prevalence countries, subdivided by country-of-origin
2. HIV-positive MSM
3. HIV-positive PWID
4. HIV-positive non-MSM non-PWID (HCV only)
5. HIV-negative MSM (HBV only)
6. HIV-negative PWID
7. Haemophilia patients treated before 1992 (HCV only)
8. Female sex workers (HBV only)
9. Individuals at low risk for infection

For HBV and HCV separately, low and high estimates of the population size and the prevalence of chronic infection within these subsets were derived from (un)published studies, national registries and personal communication with experts. MEDLINE and EMBASE databases were searched for HBV and HCV infection prevalence studies in the Netherlands that included prevalence estimates for the described risk groups and/or the general population. Bibliographies of identified studies were also searched for relevant articles. In addition, information was requested from Dutch experts working in the field of hepatitis research to obtain the most recent data from unpublished studies. A list of all selected studies was sent for review to an expert panel (consisting of medical doctors, epidemiologists, a public health nurse and a policy advisor) to ensure completeness. Chronic HBV infection refers to hepatitis B surface antigen (HBsAg) positivity. Chronic HCV infection refers to viraemic infection, i.e. HCV-RNA positivity. When studies only reported the anti-HCV antibody prevalence, this was adjusted with a correction factor of 0.74 to account for spontaneous viral clearance [22]. We estimate the number of ever-chronically-infected individuals, including those who were successfully treated. As prevalence estimates mostly refer to adult populations, we used population size estimates for the population aged 15 years and older. High and low population size estimates were multiplied by high and low estimates of HBsAg and HCV-RNA prevalences to produce estimates of the average number of individuals ever chronically infected with HBV and HCV, respectively.

### Risk group population size and prevalence

#### First-generation migrants originating from low-intermediate to high endemic countries

First-generation migrants were defined as individuals born outside of the Netherlands with at least one parent also born abroad. Second-generation migrants were not defined as risk population, because it was described that chronic viral hepatitis prevalences among them were similar to the general population [11, 12]. The numbers of first-generation migrants aged 15 years and older in 2016, stratified by country-of-origin, were obtained from Statistics Netherlands [23]. The population sizes determined by Statistics Netherlands are the most accurate estimates available; we therefore did not use a different low and high estimate of the population size per migrant group but used the Statistics Netherlands estimate for both the low and high population size estimate. All migrant groups were listed as separate risk groups by country-of-origin in the workbooks for HBV and HCV if they included at least 500 individuals and if the HBsAg and HCV-RNA prevalence estimates for these groups were at least 1.0% and 0.5% in the country-of-origin, respectively. We chose relatively low cut-off points to increase the precision of the prevalence estimations. We lowered the cut-off value for HCV to 0.5% because otherwise people born in Morocco, Turkey and Surinam (the three largest migrant populations in the Netherlands) would not be included in the analysis. Using these cut-offs, we included 79 migrant groups for HBV and 96 migrant groups for HCV. Migrant groups including less than 500 individuals (in total 22 388 migrants from 118 different countries, comprising 1.2% of all first-generation migrants) and migrants from low prevalence countries (29% and 15% of all first-generation migrants for HBV and HCV, respectively) were included in the group of individuals at low risk of infection.

For HBsAg and HCV-RNA prevalences among first-generation migrants in the Netherlands, we distinguished (1) population-based prevalence studies (i.e. participants were randomly selected from a population register) conducted in the Netherlands, which included migrants from various endemic countries and (2) screening studies performed in the Netherlands that offered screening for HBV and/or HCV to migrants from selected countries. Only screening studies including 100 or more participants were considered. If both population-based and screening studies were available for a specific country, only the results from the population-based studies were used, because these are less likely to be biased than screening studies, in which people could have participated selectively.

If no population-based or screening studies from the Netherlands were available, prevalence estimates for the country-of-origin were used as a proxy for the prevalence among migrants from that country. These estimates were derived from Schweitzer *et al*. [1] and the Polaris Observatory [2] for HBV and HCV, respectively. If also no country-specific estimates were available, regional HBV and HCV estimates (derived from Kowdley *et al*. [24] for HBV and the Polaris Observatory [2] for HCV) were used. It has previously been described that HBV prevalence estimates for the country-of-origin are often higher than the prevalence among migrants from that country, while for HCV they seem more comparable [20]. However, a Dutch study examining the HCV prevalence among migrants in the Netherlands suggested that the prevalence among migrants was lower compared with the prevalence in the country of origin [25]. To investigate whether a correction factor would be appropriate for the country-of-origin HBV and/or HCV prevalence estimates, we compared estimates derived from studies in the Netherlands with country-of-origin estimates for 10 countries for HBV and nine for HCV for which both were available. The statistical methods and results of this comparison are described in the supplementary material. For the majority of countries for which data were available the prevalence was slightly higher in the country of origin but no statistically significant differences were observed. We therefore did not use a correction factor for country-of-origin prevalence estimates. Supplementary Tables S3 (HBV) and S4 (HCV) specify which prevalence estimate sources were used for every specific country. In total, for HBV, a local estimate was available for 10 countries of origin (covering 64% of the foreign-born population) and country/region-of-origin estimate for 69. For HCV, a local estimate was available for eight (covering 42% of the foreign-born population) of 96 countries.

#### HIV-positive individuals

The Dutch Stichting HIV Monitoring (SHM, HIV Monitoring Foundation) collects longitudinal data of all newly registered HIV-infected individuals in the Netherlands and provides estimates of the size of the undiagnosed population [26]. The SHM maintains an up-to-date patient database including HBV and HCV status and risk group. We included HIV-positive MSM and HIV-positive PWID (both diagnosed and undiagnosed as estimated by the SHM) as separate risk groups. Because the majority (84%) of the non-MSM non-PWID HIV-positive individuals co-infected with HBV are of non-Western European descent (SHM, personal communication, April 2017), they were not defined as a separate risk group in the HBV workbook in order to prevent overlap with the first-generation migrant groups. On the contrary, since non-MSM non-PWID HIV-positive individuals co-infected with HCV are mostly (56%) of Dutch or Western European descent (SHM, personal communication, April 2017), this group was included as a separate risk group in the HCV workbook. The population size estimates derived from SHM were used as both low and high estimates in the workbooks. The SHM provided the prevalence of ever being diagnosed with a chronic HBV or HCV infection, stratified by transmission group.

#### HIV-negative MSM and PWID

The estimated number (low and high estimate identical) of HIV-negative MSM was derived from the estimated MSM population size in the Netherlands by Op de Coul *et al*. [27] minus the estimated number of HIV-positive MSM by SHM [26]. The HBV prevalence estimate for MSM was derived from the hepatitis B vaccination program for risk groups [28]. The estimated numbers (low and high estimates) of HIV-negative PWID were derived from estimates on the total PWID population size in the Netherlands by the Dutch Focal Point for the European Monitoring Center for Drugs and Drug Addiction [29] and the Netherlands Institute for Mental Health and Addiction [30] minus the estimated number of HIV-positive PWIDs by SHM [26]. It is important to note that PWID in the Netherlands is mostly ever-injectors rather than current-injectors and that numbers used concern opiate injectors. It is expected that the group of amphetamine and/or cocaine injectors is relatively small and do not contribute to transmission. Data from addiction care services indicate that the whole country counts less than 1000 injectors, of whom the far majority is a heroin injector. The main route of administration in crack cocaine users is basing and in amphetamine users swallowing. This information is based on expert opinions and fieldwork. HBV and HCV prevalence estimates among PWID were derived from a study among people who use drugs in The Hague, performed by the National Institute of Public Health and the Environment [31] and from the Amsterdam Cohort Studies among people who use drugs [32].

#### Haemophilia patients treated before 1992 (HCV only)

HCV is an important co-morbidity among haemophilia patients who received blood products (clotting factor concentrates) before 1992. Donor screenings for HCV were not introduced in the Netherlands until 1992 and 98% of patients ever treated before 1992 with large pool (i.e. derived from more than 1000 different donors) non-HCV-safe clotting factor concentrate was infected with HCV [33]. Low and high estimates for the number of haemophilia patients treated before 1992 who currently live in the Netherlands were derived from the Van Creveld Clinic, the largest haemophilia treatment centre in the Netherlands (Dr E. P. Mauser-Bunschoten, personal communication, April 2017) and Vriend *et al*. [34], respectively. Prevalence estimates were derived from a cross-sectional study among haemophilia patients in 2005 [35].

#### Female sex workers (HBV only)

Female sex workers were defined as sex workers working in brothels and clubs, working as escorts, or working on the street. In 1998, The European Network for HIV/STD Prevention in Prostitution (Europap) estimated that between 20 000 and 25 000 sex workers were active in the Netherlands [36]. Although dated, this is currently the best estimate. An estimated 12% of sex workers are male or transgender [37] and were deducted from the population size. HBV prevalence estimates for female sex workers were derived from surveillance data regarding HBV vaccination [38], STI clinic visitors [39] and outreach activities [39].

#### Individuals at low risk of infection

Individuals not classified as one of the aforementioned risk groups were defined as the population at low risk of infection. The low and high population size estimates were calculated by subtracting the low and high population size estimates of the risk groups from the total number of individuals aged 15 and older in the Netherlands, obtained from Statistics Netherlands [23]. In 2016, the total population in the Netherlands was almost 17 million, of which 14.2 million were aged 15 years and older. Prevalence estimates were derived from the screening of first time blood donors [40], an HCV prevalence-study among a random sample of pregnant women in Amsterdam [25], a population-based prevalence study among Amsterdam residents [41], a community-based screening project in the Rotterdam region [42] and from the national serosurvey PIENTER-2 [11, 12]. Because several studies reported an HCV-RNA prevalence of 0% among individuals at low risk of infection, this was used as the low HCV-RNA estimate.

### Statistical analysis

The prevalence estimates for each population group based on the identified studies were calculated using the workbook calculations and the upper and lower limits of the 95% confidence intervals were chosen as the high and low estimates, respectively. To obtain estimates the following procedure was applied: If multiple studies with numerator and denominator data were available, the results were pooled using meta-analyses, using the meta package in the R statistical computing environment [43]. Heterogeneity (*I*^2^) was calculated and random effects were taken into account if heterogeneity was significant; otherwise, a fixed model was chosen. Because several studies included zero-event data, a continuity correction was applied to all analyses to include these studies. Freeman-Tukey double arcsine transformations were used to calculate the overall prevalence and 95% confidence interval. If only one study was available reporting HBV or HCV prevalence (including numerator and denominator data), a 95% confidence interval was calculated using the Clopper-Pearson method with the binom package in *R.* If two or more studies were available that reported data without numerator and denominator data (e.g. only ranges) of that risk group, the highest and lowest reported estimates were used as high and low estimate in the workbook.

## Results

### Chronic HBV infections in the Netherlands

**Fig. 1.**
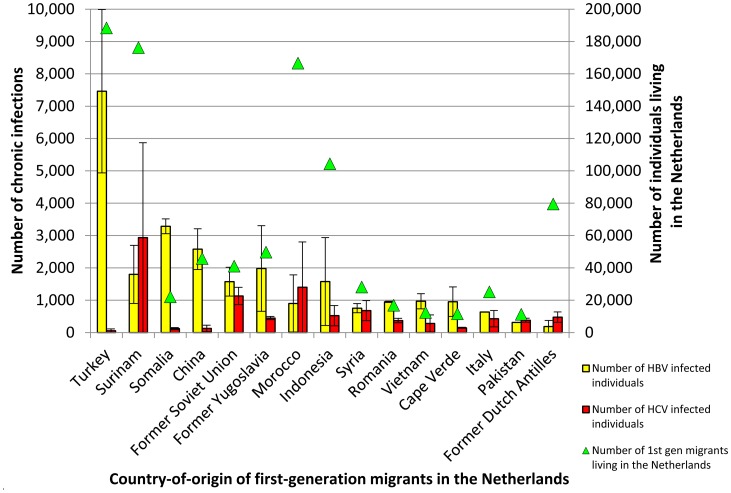
The number of chronic HBV (yellow) and ever-chronic HCV (red) infected first-generation migrants in the Netherlands shown for the top 15 migrant groups with the highest number of HBV and HCV infections combined (left *y*-axis). Triangles (green) indicate the total number of first-generation migrants living in the Netherlands for each country (right *y*-axis).

Table 1 summarizes the estimated population size and chronic HBV prevalence per population group. The overall prevalence of chronic HBV infection among individuals aged 15 and older in the Netherlands was estimated at 0.34% (low 0.22%, high 0.47%), which corresponds to 48 756 (low 31 237, high 66 461) HBV infected individuals. First-generation migrants accounted for most HBV infections (81%), with an estimated 39 521 (min 27 969, max 51 073) infected individuals. The migrant groups that were estimated to harbour most chronic HBV infections in the Netherlands are migrants from Turkey, Somalia and China with 18.9%, 8.3% and 6.5% of HBV infections among migrants, respectively (Fig. 1). Supplementary Table S3 shows the estimated population size, HBV prevalence and number of infected individuals per country-of-origin. HIV-negative MSM accounted for the second largest number of infections (1237; low 788, high 1744).

**Table 1.** Risk group specific HBsAg prevalence, population size and estimated number of chronic HBV infections

Risk group	HBsAg prevalence	Population in the Netherlands	Estimated number of chronic HBV cases
	% (low–high estimate)	(low–high estimate)	(low–high estimate)
First-generation migrants from HBV endemic countries^a^	3.08 (2.18%–3.98%)	1 284 654 (1 284 654–1 284 654)	39 521 (27 969–51 073)
HIV+ MSM	3.47 (3.13%–3.81%)	13 650 (13 200–14 100)	474 (413–537)
HIV+ PWID	4.21 (1.95%–6.46%)	511 (310–712)	21 (6–46)
HIV− MSM	0.65 (0.60%–0.70%)	190 265 (131 321–249 209)	1237 (788–1744)
HIV− PWID	3.50 (1.00%–6.00%)	5276 (4357–6194)	185 (44–372)
Female sex workers	1.14 (0.72%–1.56%)	19 800 (17 600–22 000)	226 (127–343)
Individuals at low risk of infection	0.06 (0.02%–0.10%)	12 665 193 (12 602 479–12 727 906)	7093 (1890–12 346)
*Total*	*0.34 (0.22%–0.47%)*	*14 179 348*^b^	*48 756 (31 237–66 461)*

HBsAg, Hepatitis B surface antigen; HBV, Hepatitis B virus; MSM, Men who have sex with men; PWID, People who inject drugs.

a First-generation migrants from countries with HBsAg prevalence >1.0% of which more than 500 migrants live in the Netherlands.

b Total population of the Netherlands older than 15 in 2016.

### Chronic HCV infections in the Netherlands

Table 2 summarizes the estimated population size and chronic HCV prevalence per population group. The overall prevalence of ever having been chronically infected with HCV among individuals aged 15 and older in the Netherlands was estimated at 0.16% (low 0.06%, high 0.27%). This corresponds to 22 885 (low 8461, high 37 809) individuals. Concordant with HBV, first-generation migrants comprise the largest group of HCV infected individuals (60%), with 13 819 (low 5671, high 21 967) ever chronically infected individuals. The migrant groups that were estimated to harbour most chronic HCV infections in the Netherlands are migrants from Surinam, Morocco and the former Soviet Union with 21.2%, 10.1% and 8.2% of HBV infections among migrants, respectively (Fig. 1). Supplementary Table S4 shows the estimated population size and HCV prevalence per country-of-origin. HIV-negative PWID are the second largest group with an estimated 3131 (min 1522, max 5189) ever chronically infected individuals. Figure 1 shows the first-generation migrant groups with the highest absolute number of chronic HBV and HCV infected individuals for the countries of origin with the largest number of (combined) infections.

**Table 2.** Risk group specific HCV-RNA prevalence, population size and estimated number of chronic HCV infections

Risk group	HCV-RNA prevalence	Population in the Netherlands	Estimated number of chronic HCV cases
	% (low–high estimate)	(low–high estimate)	(low–high estimate)
First-generation migrants from HCV endemic countries^a^	0.90 (0.37%–1.44%)	1 527 032 (1 527 032–1 527 032)	13 819 (5671–21 967)
HIV+ MSM	4.92 (4.55%–5.29%)	13 650 (13 200–14 100)	672 (601–746)
HIV+ PWID	59.35 (55.70%–63.00%)	511 (310–712)	303 (173–449)
HIV+ Non-PWID Non-MSM	3.68 (3.26%–4.10%)	8889 (8289–9489)	327 (270–389)
HIV− PWID	59.36 (34.93%– 83.78%)	5276 (4357–6194)	3131 (1522–5189)
Haemophilia patients treated before 1992	53.90 (49.96%–57.84%)	785 (450–1120)	423 (225–648)
Individuals at low risk of infection	0.03 (0.00%–0.07%)	12 623 206 (12 620 701–12 625 710)	4210 (0–8421)
*Total*	*0.16 (0.06%–0.27%)*	*14 179 348*^b^	*22 885 (8461–37 809)*

HCV, Hepatitis C virus; RNA, Ribonucleic acid; MSM, Men who have sex with men; PWID, People who inject drugs.

a First-generation migrants from countries with HCV-RNA prevalence >0.5% of which more than 500 migrants live in the Netherlands.

b Total population of the Netherlands older than 15 in 2016.

## Discussion

The chronic HBV prevalence of 0.34% in the Netherlands is among the lowest prevalences in Europe [1] and the chronic HCV prevalence of 0.16% is among the lowest in the world [2]. Detailed insight in the prevalence and estimated number of people with chronic infection in specific risk group is given, for first-generation migrants by country of birth. First-generation migrants account for most infections with 81% of HBV and 60% of HCV infections.

Recently, WHO published their global strategy on viral hepatitis 2016–2021 [9], in which they give directions to getting to know the epidemic in the country in order to tailor health investments well. Furthermore, in the 2017 Global Hepatitis Report they reported that, while global estimates are taking shape, key data remains missing in many countries [44]. Our method could serve as an example of an accessible method to estimate risk group specific and overall HBV/HCV prevalences.

Our study is, however, subject to several limitations. Firstly, it should be noted that several relatively small risk groups (e.g. haemodialysis patients, HIV-negative MSM using pre-exposure prophylaxis (PrEP) [45] and asylum seekers or undocumented migrants) were not specifically included in our calculations. Due to their limited size (e.g. due to the severely lower life expectancy a limited number of haemodialysis patients treated before 1992 is expected to be currently alive, only 376 MSM were participating in a PrEP demonstration project in the Netherlands is 2016 [45]), we do not expect this to majorly influence the overall prevalence. First-generation migrant children under the age of 15 were also not included. We decided not to include this group because the prevalence data available from Dutch studies among migrants did not include children. As the prevalence among children is expected to be lower, applying adult prevalence estimates to the total population including children would have resulted in an overestimation of the population with chronic viral hepatitis. Secondly, although we attempted to use the best available data, some data were subject to different types of bias that possibly influenced the outcomes. Prevalence estimates for haemophilia patients and female sex workers were derived from studies conducted 10–15 years ago and may no longer be accurate. However, they are, to date, the best source available. In addition, we combine data sources that use different methodologies to estimate population sizes (i.e. for HIV-negative MSM). These numbers may not be fully compatible and should be viewed with some caution. Prevalence estimates derived from studies among first-generation migrants in the Netherlands were available for 64% and 42% of the migrant population for HBV and HCV, respectively. For the remaining migrants, prevalence estimates were derived from studies in the countries of origin. As we showed in the supplementary material, for some countries these country-of-origin data may overestimate the prevalence among migrants. This may be explained by a lower risk of exposure after migration to a lower prevalence country and epidemiological phenomena such as the healthy migrant effect or salmon bias [46]. Furthermore, HBV prevalences for nine countries and HCV prevalences for six countries were derived from targeted screening studies rather than population-based studies. In a separate analysis (data not shown), we tested for differences between prevalence estimates from screening and population-based studies among Turkish migrants for which three studies of both types were available. We found a significant lower HBV prevalence in screening studies among Turkish migrants than in population-based studies (2.54% *vs.* 4.11%, *p* < 0.05). This suggests, for HBV among Turkish migrants at least, that prevalence estimates from screening studies may underestimate the prevalence in the population. A further limitation is that we estimated the total number of people who ever had, or currently have, a chronic HCV infection. The prevalence of current chronic HCV infection will be slightly lower as individuals successfully treated (estimated at 4427 from 2009 to 2015, of which 2000 successfully treated with DAAs in 2015) have lost their HCV RNA [47]. Unfortunately, data on diagnoses and treatment outcomes are not available at the risk group level in the Netherlands, except for HIV-infected individuals.

Our chronic HBV prevalence estimate is lower than a previous study by Marschall *et al*. in 2006 [48], that used country-of-birth prevalence data for the migrant groups, which might be an overestimation, at least for some countries, as we showed in our analyses. Another explanation for the lower prevalence we found might be the reported global decrease in the number of persons with chronic HBV infection in the past decades [1]. Our chronic HCV prevalence estimate is concordant with a previous workbook estimate by Vriend *et al*. [34]. However, a recent modelling study by Razavi *et al*. [49] estimated the chronic HCV prevalence in the Netherlands in 2015 at 0.11% (95% uncertainty interval 0.03–0.15), which is slightly lower than our estimate but within our range of 0.06–0.27. This may be explained by the fact that they included persons aged <15 years who are at lower risk of infection and took treatment into account, both lowering the overall prevalence. Their model, however, does not discriminate between the different risk groups, leaving the question of how the infections are distributed in the population unanswered.

The finding that migrants account for most HBV and HCV infections is concordant with findings from two earlier studies performed in the Netherlands [34, 48]. The disproportional burden of chronic viral hepatitis among migrants stresses the importance of developing and implementing targeted screening interventions directed at first-generation migrant populations for whom – unlike for other risk groups in the Netherlands – no structural screening programs are in place (besides antenatal HBV screening of pregnant women). Because the heterogeneity of the migrant population is high in terms of ethnic origin, risk for infection and awareness of infection, different screening approaches may be needed. Good practice examples have been described, including a toolkit developed by the ‘HEPscreen’ project (http://hepscreen.eu/). Our study shows that combining population size and prevalence estimates per migrant group gives insight in which migrant groups the highest number of chronic infections are expected and shows the large differences between countries of origin and between HBV and HCV. For example, the largest first-generation migrant population from hepatitis B or C endemic countries in the Netherlands is originating from Turkey (Fig. 1). While the intermediate HBV prevalence of 4% translates to a high number of chronic HBV infections, the expected number of HCV infections among Turkish migrants is very low. In contrast, migrants from the former Soviet Union are a smaller population but the relatively high HBV and HCV prevalence indicate that combined screening for both infections would be justified. An economic evaluation is needed to gain insight in the conditions when combined HBV/HCV screening would be cost-effective compared with screening for HBV or HCV alone, or no screening. Some screening interventions in the Netherlands have targeted different migrant groups [14, 16, 18, 19, 42]. These interventions, however, were once-off, limited in geographical coverage and uptake was often low/moderate depending on the screening model. A systematic review of the outcomes of hepatitis C screening programs worldwide concluded that HCV screening programs in the past identified only a small portion of the estimated number of HCV-infected individuals [50]. Systematic screening programs for migrants are needed to substantially reduce chronic viral hepatitis-related morbidity and mortality among migrants and the sustainability, linkage to care and access to treatment has to be ensured.

Besides the evidently beneficial screening of risk groups, it will be an even bigger challenge to identify the individuals in the ‘low-risk’ group. These individuals are hidden in the general population but do account for 15% and 18% of HBV and HCV infections, respectively. A birth-cohort screening approach, which was piloted in the south of the Netherlands, was unsuccessful in identifying undiagnosed infections [51]. Vigilance of health care providers, for example to prompt viral hepatitis testing when people present with abnormal liver function, is needed to identify those infected who do not belong to one of the risk groups.

In conclusion, the prevalence of both chronic HBV and HCV infection in the Netherlands is low and migrants account for most infections. The risk group-specific prevalence estimates, for first-generation migrants stratified by country of birth, provide insight into the distribution of chronic infections. Outcomes can be used to implement screening effectively and monitor progress towards the elimination of chronic viral hepatitis.

## Supplementary material

For supplementary material accompanying this paper visit https://doi.org/10.1017/S0950268819000359. click here to view supplementary material
