# Supplementary material for: Chronic hepatitis B and C infections in the Netherlands: estimated prevalence in risk groups and the general population
Source: Epidemiol Infect. 2019 Mar 14;147:e147. doi: 10.1017/S0950268819000359 (PMC6518512; doi:10.1017/S0950268819000359)
Supplement: Supplementary file 1 [file S0950268819000359sup001.docx]

**SUPPLEMENTARY MATERIAL**

Epidemiology and Infection

Manuscript: Chronic hepatitis B and C infections in the Netherlands: estimated prevalence in high-risk groups and the general population

J. Koopsen, J.E. van Steenbergen, J.H. Richardus, M. Prins, E.L.M. Op de Coul, E.A. Croes, J. Heil, F.R. Zuure and I.K. Veldhuijzen.

Contents

[1. Comparison of prevalence estimates from studies among migrants in the Netherlands to country-of-origin data 1](#_Toc493593776)

[Table S1. HBV prevalences derived from studies among first-generation migrants in the Netherlands compared to country-of-origin HBV prevalence estimates. 2](#_Toc493593777)

[Table S2. HCV prevalences derived from studies among first-generation migrants in the Netherlands compared to country-of-origin HCV prevalence estimates. 2](#_Toc493593778)

[2. Chronic HBV and HCV prevalences and cases among first generation migrants in the Netherlands, stratified by country of origin 4](#_Toc493593779)

[Table S3. Chronic HBV prevalences and cases among first generation migrants in the Netherlands, stratified by country-of-origin. 4](#_Toc493593780)

[Table S4. Chronic HCV prevalences and cases among first generation migrants in the Netherlands, stratified by country-of-origin. 6](#_Toc493593781)

[3. Supplement references 8](#_Toc493593782)

# Comparison of prevalence estimates from studies among migrants in the Netherlands to country-of-origin data

*Methods*

To test for differences between prevalence estimates derived from studies among first-generation migrants in the Netherlands and country-of-origin prevalence estimates, ratios between proportions were determined using Monte Carlo simulation generating 10,000 realizations with the significance level set at p<0.05. As the country-of-origin prevalences derived from Schweitzer et al. [1] and the Polaris observatory [2] were only available as proportions with 95% confidence intervals, while prevalences derived from studies conducted in the Netherlands were available as proportions with numerator and denominator data, two approaches were used to generate the 10,000 realizations: a beta distribution was fitted to the proportions of which numerator and denominator data were available [3] and a three-parameter skew-normal distribution was fitted (after a logit-transformation) to proportions with a 95% CI [4]. The skew-normal distribution was used because proportions with a 95% CI could not be described using a beta distribution.

*Results*For six out of the ten countries with HBV prevalence data from both Dutch studies and studies in the countries of origin, no significant differences were found. Four out of the ten studies reported significantly lower HBV prevalences among first-generation migrants in the Netherlands compared with the estimated HBV prevalence in the country of origin (Table S1). For six out of the nine countries of which HCV prevalences were available from both Dutch studies and global modeling studies, no significant difference was found. Out of the nine countries, three exhibited a significantly lower HCV prevalence among first-generation migrants in the Netherlands than in the country of origin (Table S2).

## Table S1. HBV prevalences derived from studies among first-generation migrants in the Netherlands compared to country-of-origin HBV prevalence estimates.

| Country | HBV estimate based on studies among migrants in the Netherlands | Country-of-origin HBV estimate | Ratio* | p-value | Source in Dutch migrants | Source in country |
| --- | --- | --- | --- | --- | --- | --- |
| China | 5.74% | 5.49% | 0.96 | 0.432 | [5, 6] | [1] |
| Dutch Antilles | 0.46% | 4.52% | 29.76 | < 0.001 | [7, 8] | [1] |
| Egypt | 1.30% | 1.71% | 1.56 | 0.380 | [9, 10] | [1] |
| Morocco | 0.68% | 1.09% | 1.96 | 0.179 | [8, 11, 12] | [1] |
| Turkey | 4.11% | 4.00% | 1.00 | 0.920 | [8, 11, 12] | [1] |
| Vietnam | 7.77% | 10.79% | 1.41 | 0.004 | [13, 14] | [1] |
| Surinam | 1.04% | 3.95% | 4.04 | < 0.001 | [7, 12] | [1] |
| Indonesia | 0.98% | 1.86% | 3.17 | 0.176 | [7] | [1] |
| Afghanistan | 2.22% | 1.63% | 0.86 | 0.539 | [13] | [1] |
| Ghana | 5.31% | 12.89% | 2.52 | < 0.001 | [12] | [1] |

* Ratio is calculated as average ratio from 10,000 random Monte Carlo realizations modelled to the study data (see methods). Hence the ratio is often not equivalent to country-of-origin estimate divided by the estimate in the Netherlands.

## Table S2. HCV prevalences derived from studies among first-generation migrants in the Netherlands compared to country-of-origin HCV prevalence estimates.

| Country | HCV estimate based on studies among migrants in the Netherlands | Country-of-origin HCV estimate | Ratio* | p-value | Source in Dutch migrants | Source  in country |
| --- | --- | --- | --- | --- | --- | --- |
| China | 0.27% | 0.66% | 2.44 | 0.01 | [6, 15, 16] | [2] |
| Egypt | 2.10% | 5.84% | 2.79 | <0.001 | [9, 10] | [2] |
| Morocco | 0.39% | 0.72% | 1.85 | 0.178 | [8, 12, 17] | [2] |
| Surinam | 0.59% | 0.56% | 0.96 | 0.997 | [8, 12, 17] | [2] |
| Turkey | 0.05% | 0.61% | 11.43 | 0.007 | [8, 12, 17] | [2] |
| Vietnam | 1.20% | 0.94% | 0.79 | 0.974 | [13] | [2] |
| Iran | 0.32% | 0.23% | 0.72 | 0.807 | [13] | [2] |
| Iraq | 0.17% | 0.23% | 1.35 | 0.499 | [13] | [2] |
| Afghanistan | 0.17% | 0.51% | 3.07 | 0.179 | [13] | [2] |

* Ratio is calculated as average ratio from 10,000 random Monte Carlo realizations modelled to the study data (see methods). Hence the ratio is often not equivalent to country-of-origin estimate divided by the estimate in the Netherlands.

# Chronic HBV and HCV prevalences and cases among first generation migrants in the Netherlands, stratified by country of origin

## Table S3. Chronic HBV prevalences and cases among first generation migrants in the Netherlands, stratified by country-of-origin.

| Country | First-generation migrants living in the Netherlands, 2016* | HBV prevalence  (Low - High estimate) | HBV cases  (Low - High estimate) | Source(s) |
| --- | --- | --- | --- | --- |
|  |  |  |  |  |
| Afghanistan | 31,998 | 2.67% (0.94% - 4.40%) | 855 (301 - 1409) | [13] |
| Albania | 1,502 | 7.80% (7.56% - 8.03%) | 117 (114 - 121) | [1] |
| Algeria | 3,829 | 2.92% (2.50% - 3.33%) | 112 (96 - 128) | [1] |
| Angola | 4,803 | 12.49% (11.09% - 13.88%) | 600 (533 - 667) | [1] |
| Austria | 5,371 | 1.34% (0.81% - 1.86%) | 72 (44 - 100) | [1] |
| Azerbaijan | 717 | 3.10% (1.71% - 4.49%) | 22 (12 - 32) | [1] |
| Bangladesh | 933 | 3.10% (2.99% - 3.21%) | 29 (28 - 30) | [1] |
| Bosnia and Herzegovina | 684 | 1.13% (0.91% - 1.35%) | 8 (6 - 9) | [1] |
| Bulgaria | 19,742 | 4.00% (3.19% - 4.81%) | 790 (630 - 950) | [1] |
| Burundi | 2,029 | 9.85% (5.97% - 13.73%) | 200 (121 - 279) | [1] |
| Cambodia | 686 | 4.08% (3.57% - 4.59%) | 28 (24 - 31) | [1] |
| Cameroon | 1,639 | 12.25% (11.71% - 12.78%) | 201 (192 - 209) | [1] |
| Cape Verde | 11,655 | 8.18% (4.26% - 12.10%) | 953 (497 - 1410) | [1] |
| China | 45,842 | 5.63% (4.25% - 7.00%) | 2,579 (1,948 - 3,209) | [5, 6] |
| Colombia | 8,811 | 2.34% (1.86% - 2.82%) | 206 (164 - 248) | [1] |
| Congo | 845 | 11.02% (9.75% - 12.29%) | 93 (82 - 104) | [1] |
| Congo (DR) | 4,465 | 6.00% (5.68% - 6.31%) | 268 (254 - 282) | [1] |
| Cuba | 1,257 | 1.66% (0.62% - 2.70%) | 21 (8 - 34) | [1] |
| Cyprus | 560 | 2.71% (2.38% - 3.04%) | 15 (13 - 17) | [1] |
| Dominican Republic | 8,291 | 4.45% (2.65% - 6.25%) | 369 (220 - 518) | [1] |
| Ecuador | 2,140 | 2.38% (1.08% - 3.68%) | 51 (23 - 79) | [1] |
| Egypt | 11,936 | 0.95% (0.04% - 1.85%) | 113 (5 - 221) | [9, 10] |
| Eritrea | 6,492 | 2.50% (2.32% - 2.67%) | 162 (151 - 173) | [1] |
| Ethiopia | 10,961 | 6.04% (5.77% - 6.31%) | 662 (632 - 692) | [1] |
| Former Dutch Antilles | 79,574 | 0.24% (0.00% - 0.47%) | 187 (0 - 374) | [7, 8] |
| Former Soviet Union | 41,123 | 3.83% (2.74% - 4.91%) | 1,573 (1,127 - 2,019) | [18] (Regional) |
| Former Yugoslavia | 49,784 | 3.98% (1.32% - 6.64%) | 1,981 (657 - 3,306) | [18] |
| Gambia | 687 | 12.30% (11.50% - 13.09%) | 84 (79 - 90) | [1] |
| Ghana | 13,338 | 5.55% (3.58% - 7.52%) | 740 (478 - 1,003) | [12] |
| Guinea | 2,311 | 15.09% (14.16% - 16.01%) | 349 (327 - 370) | [1] |
| Guyana | 2,245 | 1.32% (0.72% - 1.91%) | 30 (16 - 43) | [18] |
| Hong Kong | 9,617 | 8.98% (8.47% - 9.48%) | 863 (815 - 912) | (18) (Regional) |
| India | 21,848 | 1.46% (1.44% - 1.47%) | 318 (315 - 321) | [1] |
| Indonesia | 104,480 | 1.51% (0.21% - 2.81%) | 1,578 (219 - 2,936) | [7] |
| Italy | 25,242 | 2.52% (2.49% - 2.54%) | 635 (629 - 641) | [1] |
| Ivory Coast | 1,049 | 9.42% (8.70% - 10.14%) | 99 (91 - 106) | [1] |
| Jamaica | 932 | 3.97% (2.65% - 5.29%) | 37 (25 - 49) | [1] |
| Japan | 5,076 | 1.02% (1.01% - 1.02%) | 52 (51 - 52) | [1] |
| Jordan | 856 | 1.87% (1.68% - 2.06%) | 16 (14 - 18) | [1] |
| Kenya | 2,046 | 5.17% (4.86% - 5.48%) | 106 (99 - 112) | [1] |
| Latvia | 3,273 | 1.39% (1.10% - 1.67%) | 45 (36 - 55) | [18] |
| Lebanon | 3,057 | 1.22% (1.10% - 1.34%) | 37 (34 - 41) | [1] |
| Liberia | 1,575 | 17.63% (15.70% - 19.55%) | 278 (247 - 308) | [1] |
| Libya | 1,063 | 2.16% (2.05% - 2.27%) | 23 (22 - 24) | [1] |
| Lithuania | 4,563 | 1.71% (1.55% - 1.86%) | 78 (71 - 85) | [1] |
| Morocco | 166,727 | 0.54% (0.01% - 1.07%) | 900 (17 - 1,784) | [8, 11, 12] |
| Mozambique | 564 | 8.38% (7.55% - 9.21%) | 47 (43 - 52) | [1] |
| Myanmar | 1,061 | 3.40% (3.26% - 3.54%) | 36 (35 - 38) | [1] |
| New Zealand | 1,877 | 4.11% (4.04% - 4.18%) | 77 (76 - 78) | [1] |
| Nigeria | 6,016 | 9.76% (9.59% - 9.93%) | 587 (577 - 597) | [1] |
| Pakistan | 11,395 | 2.76% (2.73% - 2.79%) | 315 (311 - 318) | [1] |
| Peru | 3,503 | 2.11% (1.90% - 2.32%) | 74 (67 - 81) | [1] |
| Philippines | 11,737 | 4.63% (4.53% - 4.73%) | 543 (532 - 555) | [1] |
| Portugal | 15,681 | 1.05% (0.78% - 1.31%) | 164 (122 - 205) | [1] |
| Romania | 16,936 | 5.62% (5.50% - 5.73%) | 951 (931 - 970) | [1] |
| Russia | 2,279 | 2.74% (2.64% - 2.83%) | 62 (60 - 64) | [1] |
| Rwanda | 983 | 7.60% (3.82% - 11.37%) | 75 (38 - 112) | [1] |
| Saudi Arabia | 1,615 | 3.18% (3.12% - 3.24%) | 51 (50 - 52) | [1] |
| Senegal | 894 | 11.06% (10.72% - 11.40%) | 99 (96 - 102) | [1] |
| Sierra Leone | 3,731 | 8.86% (5.99% - 11.73%) | 331 (223 - 438) | [1] |
| Singapore | 2,530 | 4.10% (3.87% - 4.33%) | 104 (98 - 110) | [1] |
| Somalia | 22,189 | 14.81% (13.77% - 15.84%) | 3,285 (3,055 - 3,515) | [1] |
| South Africa | 8,857 | 6.70% (6.56% - 6.83%) | 593 (581 - 605) | [1] |
| South Korea | 3,497 | 4.37% (4.36% - 4.37%) | 153 (152 - 153) | [1] |
| Sri Lanka | 6,755 | 2.61% (1.90% - 3.31%) | 176 (128 - 224) | [1] |
| Sudan | 4,342 | 9.79% (9.03% - 10.54%) | 425 (392 - 458) | [1] |
| Suriname | 176,284 | 1.02% (0.51% - 1.53%) | 1,798 (899 - 2,697) | [7, 12] |
| Syria | 28,254 | 2.67% (2.17% - 3.17%) | 754 (613 - 896) | [1] |
| Taiwan | 2,384 | 12.25% (11.70% - 12.80%) | 292 (279 - 305) | [18] |
| Tanzania | 919 | 7.19% (6.59% - 7.79%) | 66 (61 - 72) | [1] |
| Thailand | 12,118 | 6.42% (6.37% - 6.47%) | 778 (772 - 784) | [1] |
| Togo | 1,108 | 11.52% (7.45% - 15.59%) | 128 (83 - 173) | [1] |
| Tunisia | 4,317 | 6.18% (5.95% - 6.40%) | 267 (257 - 276) | [1] |
| Turkey | 188,450 | 3.96% (2.62% - 5.30%) | 7,463 (4,937 - 9,988) | [8, 11, 12] |
| Uganda | 1,426 | 9.21% (8.65% - 9.77%) | 131 (123 - 139) | [1] |
| Ukraine | 1,169 | 1.50% (1.10% - 1.89%) | 17 (13 - 22) | [1] |
| Vietnam | 12,539 | 7.72% (5.86% - 9.58%) | 968 (735 - 1,201) | [13, 14] |
| Zambia | 593 | 6.10% (5.38% - 6.82%) | 36 (32 - 40) | [1] |

* Number of first-generation migrants living in the Netherlands derived from Statistics Netherlands (http://statline.cbs.nl).
 HBV = hepatitis B virus, DR = Democratic Republic.

(Continued)

## Table S4. Chronic HCV prevalences and cases among first generation migrants in the Netherlands, stratified by country-of-origin.

| Country | First-generation migrants living in the Netherlands, 2016* | Viraemic HCV prevalence (Low - High estimate) | Viraemic HCV cases (Low - High estimate) | Source(s) |
| --- | --- | --- | --- | --- |
|  |  |  |  |  |
| Afghanistan | 31,998 | 0.65% (0.00% - 1.29%) | 206 (0 - 413) | [13] |
| Albania | 1,502 | 0.90% (0.80% - 1.00%) | 14 (12 - 15) | [2] (Regional) |
| Algeria | 3,829 | 1.00% (0.30% - 1.70%) | 38 (11 - 65) | [2] |
| Angola | 4,803 | 3.50% (0.10% - 6.90%) | 168 (5 - 331) | [2], (Regional) |
| Argentina | 2,946 | 0.75% (0.30% - 1.20%) | 22 (9 - 35) | [2] |
| Armenia | 675 | 3.35% (2.80% - 3.90%) | 23 (19 - 26) | [2] (Regional) |
| Australia | 5,112 | 0.85% (0.70% - 1.00%) | 43 (36 - 51) | [2] |
| Azerbaijan | 717 | 1.70% (1.30% - 2.10%) | 12 (9 - 15) | [2] |
| Bangladesh | 933 | 0.95% (0.20% - 1.70%) | 9 (2 - 16) | [19] |
| Belgium | 37,204 | 0.45% (0.20% - 0.70%) | 167 (74 - 260) | [2] |
| Bolivia | 691 | 0.45% (0.30% - 0.60%) | 3 (2 - 4) | [2] (Regional) |
| Bosnia and Herzegovina | 684 | 0.90% (0.80% - 1.00%) | 6 (5 - 7) | [2] (Regional) |
| Brazil | 13,715 | 0.75% (0.60% - 0.90%) | 103 (82 - 123) | [2] |
| Bulgaria | 19,742 | 1.15% (0.70% - 1.60%) | 227 (138 - 316) | [2] |
| Burundi | 2,029 | 2.40% (0.80% - 4.00%) | 49 (16 - 81) | [2] |
| Cambodia | 686 | 1.30% (0.90% - 1.70%) | 9 (6 - 12) | [2] |
| Cameroon | 1,639 | 0.65% (0.50% - 0.80%) | 11 (8 - 13) | [2] |
| Canada | 5,306 | 0.55% (0.40% - 0.70%) | 29 (21 - 37) | [2] |
| Cape Verde | 11,655 | 1.25% (1.10% - 1.40%) | 146 (128 - 163) | [2] (Regional) |
| China | 45,842 | 0.28% (0.06% - 0.50%) | 128 (28 - 229) | [6, 15, 16] |
| Colombia | 8,811 | 0.75% (0.60% - 0.90%) | 66 (53 - 79) | [2] |
| Congo | 845 | 3.50% (0.10% - 6.90%) | 30 (1 - 58) | [2] (Regional) |
| Congo (DR) | 4,465 | 3.50% (0.10% - 6.90%) | 156 (4 - 308) | [2] (Regional) |
| Costa Rica | 579 | 0.45% (0.40% - 0.50%) | 3 (2 - 3) | [2] (Regional) |
| Dominican Republic | 8,291 | 0.70% (0.40% - 1.00%) | 58 (33 - 83) | [2] |
| Ecuador | 2,140 | 0.45% (0.30% - 0.60%) | 10 (6 - 13) | [2] (Regional) |
| Egypt | 11,936 | 1.66% (0.43% - 2.89%) | 198 (51 - 345) | [9, 10] |
| Eritrea | 6,492 | 0.55% (0.40% - 0.70%) | 36 (26 - 45) | [2], (Regional) |
| Estonia | 930 | 1.25% (0.90% - 1.60%) | 12 (8 - 15) | [2] |
| Ethiopia | 10,961 | 0.55% (0.40% - 0.70%) | 60 (44 - 77) | [2] |
| Former Dutch Antilles | 79,574 | 0.60% (0.40% - 0.80%) | 477 (318 - 637) | [2] (Regional) |
| Former Soviet Union | 41,123 | 2.75% (2.10% - 3.40%) | 1,131 (864 - 1,398) | [2] (Regional) |
| Former Yugoslavia | 49,784 | 0.90% (0.80% - 1.00%) | 448 (398 - 498) | [2] (Regional) |
| Ghana | 13,338 | 0.38% (0.00% - 0.76%) | 51 (0 - 101) | [12] |
| Greece | 13,240 | 1.10% (0.70% - 1.50%) | 146 (93 - 199) | [2] |
| Guinea | 2,311 | 1.25% (1.10% - 1.40%) | 29 (25 - 32) | [2] (Regional) |
| Guyana | 2,245 | 0.60% (0.40% - 0.80%) | 13 (9 - 18) | [2] (Regional) |
| Hungary | 12,871 | 0.45% (0.30% - 0.60%) | 58 (39 - 77) | [2] |
| India | 21,848 | 0.60% (0.40% - 0.80%) | 131 (87 - 175) | [2] |
| Indonesia | 104,480 | 0.50% (0.20% - 0.80%) | 522 (209 - 836) | [2] |
| Ireland | 4,770 | 0.65% (0.40% - 0.90%) | 31 (19 - 43) | [2] |
| Israel | 4,743 | 1.00% (0.70% - 1.30%) | 47 (33 - 62) | [2] |
| Italy  (Continued) | 25,242 | 1.70% (0.70% - 2.70%) | 429 (177 - 682) | [2] |
| Ivory Coast | 1,049 | 1.25% (1.10% - 1.40%) | 13 (12 - 15) | [2] (Regional) |
| Jamaica | 932 | 0.60% (0.40% - 0.80%) | 6 (4 - 7) | [2] (Regional) |
| Japan | 5,076 | 0.55% (0.30% - 0.80%) | 28 (15 - 41) | [2] |
| Kuwait | 1,217 | 1.65% (1.40% - 1.90%) | 20 (17 - 23) | [2] (Regional) |
| Latvia | 3,273 | 2.00% (1.40% - 2.60%) | 65 (46 - 85) | [2] |
| Liberia | 1,575 | 1.25% (1.10% - 1.40%) | 20 (17 - 22) | [2] (Regional) |
| Libya | 1,063 | 0.60% (0.50% - 0.70%) | 6 (5 - 7) | [2] |
| Lithuania | 4,563 | 1.00% (0.70% - 1.30%) | 46 (32 - 59) | [2] |
| Luxembourg | 747 | 0.80% (0.60% - 1.00%) | 6 (4 - 7) | [2] |
| Malaysia | 3,017 | 1.05% (0.80% - 1.30%) | 32 (24 - 39) | [2] |
| Morocco | 166,727 | 0.84% (0.00% - 1.68%) | 1401 (0 - 2,801) | [8, 12, 17] |
| Mozambique | 564 | 0.55% (0.40% - 0.70%) | 3 (2 - 4) | [2] (Regional) |
| Myanmar | 1,061 | 0.65% (0.50% - 0.80%) | 7 (5 - 8) | [2] (Regional) |
| Nepal | 1,475 | 1.00% (0.70% - 1.30%) | 15 (10 - 19) | [2] (Regional) |
| New Zealand | 1,877 | 0.95% (0.60% - 1.30%) | 18 (11 - 24) | [2] |
| Nigeria | 6,016 | 1.20% (1.00% - 1.40%) | 72 (60 - 84) | [2] |
| Pakistan | 11,395 | 3.35% (2.80% - 3.90%) | 382 (319 - 444) | [2] |
| Peru | 3,503 | 0.45% (0.30% - 0.60%) | 16 (11 - 21) | [2] |
| Philippines | 11,737 | 0.45% (0.30% - 0.60%) | 53 (35 - 70) | [2] |
| Poland | 107,919 | 0.50% (0.40% - 0.60%) | 540 (432 - 648) | [2] |
| Portugal | 15,681 | 0.90% (0.70% - 1.10%) | 141 (110 - 172) | [2] |
| Romania | 16,936 | 2.20% (1.80% - 2.60%) | 373 (305 - 440) | [2] |
| Russia | 2,279 | 2.90% (2.30% - 3.50%) | 66 (52 - 80) | [2] |
| Rwanda | 983 | 0.55% (0.40% - 0.70%) | 5 (4 - 7) | [2] (Regional) |
| Senegal | 894 | 1.25% (1.10% - 1.40%) | 11 (10 - 13) | [2] (Regional) |
| Serbia | 721 | 0.90% (0.80% - 1.00%) | 6 (6 - 7) | [2] (Regional) |
| Sierra-Leone | 3,731 | 1.25% (1.10% - 1.40%) | 47 (41 - 52) | [2] (Regional) |
| Singapore | 2,530 | 0.50% (0.30% - 0.70%) | 13 (8 - 18) | [2] (Regional) |
| Somalia | 22,189 | 0.55% (0.40% - 0.70%) | 122 (89 - 155) | [2] (Regional) |
| South Africa | 8,857 | 0.65% (0.40% - 0.90%) | 58 (35 - 80) | [2] |
| South Korea | 3,497 | 0.40% (0.30% - 0.50%) | 14 (10 - 17) | [2] |
| Spain | 21,806 | 0.75% (0.30% - 1.20%) | 164 (65 - 262) | [2] |
| Sri Lanka | 6,755 | 0.65% (0.50% - 0.80%) | 44 (34 - 54) | [2] (Regional) |
| Sudan | 4,342 | 0.55% (0.40% - 0.70%) | 24 (17 - 30) | [2] (Regional) |
| Suriname | 176,284 | 1.67% (0.00% - 3.33%) | 2,935 (0 - 5,870) | [8, 12, 17] |
| Switzerland | 4,660 | 0.85% (0.60% - 1.10%) | 40 (28 - 51) | [2] |
| Syria | 28,254 | 2.40% (1.30% - 3.50%) | 678 (367 - 989) | [2] |
| Taiwan | 2,384 | 2.50% (1.30% - 3.70%) | 60 (31 - 88) | [2] |
| Tanzania | 919 | 0.55% (0.40% - 0.70%) | 5 (4 - 6) | [2] (Regional) |
| Thailand | 12,118 | 0.55% (0.40% - 0.70%) | 67 (48 - 85) | [2] |
| The Gambia | 687 | 0.90% (0.50% - 1.30%) | 6 (3 - 9) | [2] |
| Togo | 1,108 | 1.25% (1.10% - 1.40%) | 14 (12 - 16) | [2] (Regional) |
| Trinidad and Tobago | 502 | 0.60% (0.40% - 0.80%) | 3 (2 - 4) | [2] (Regional) |
| Tunisia | 4,317 | 0.65% (0.20% - 1.10%) | 28 (9 - 47) | [2] |
| Turkey | 188,450 | 0.03% (0.00% - 0.06%) | 57 (0 - 113) | [8, 12, 17] |
| Uganda  (Continued) | 1,426 | 0.55% (0.40% - 0.70%) | 8 (6 - 10) | [2] (Regional) |
| Ukraine | 1,169 | 2.75% (2.10% - 3.40%) | 32 (25 - 40) | [2] (Regional) |
| United Arab Emirates | 547 | 1.05% (0.50% - 1.60%) | 6 (3 - 9) | [2] |
| Uruguay | 589 | 0.60% (0.30% - 0.90%) | 4 (2 - 5) | [2] (Regional) |
| USA | 20,775 | 0.95% (0.70% - 1.20%) | 197 (145 - 249) | [2] |
| Vietnam | 12,539 | 2.25% (0.14% - 4.35%) | 282 (18 - 545) | [13] |
| Zambia | 593 | 0.55% (0.40% - 0.70%) | 3 (2 - 4) | [2] (Regional) |

* Number of first-generation migrants living in the Netherlands derived from Statistics Netherlands (http://statline.cbs.nl).
 HCV = hepatitis C virus, DR = Democratic Republic.

# Supplement references

1. **Schweitzer A, et al.** Estimations of worldwide prevalence of chronic hepatitis B virus infection: a systematic review of data published between 1965 and 2013. *Lancet* 2015; **386**(10003): 1546-1555.

2. **Polaris Observatory HCV Collaborators.** Global prevalence and genotype distribution of hepatitis C virus infection in 2015: a modelling study. *The Lancet Gastroenterology & Hepatology* 2017; **2**(3): 161-176.

3. **Cheng RC.** Generating beta variates with nonintegral shape parameters. *Communications of the ACM* 1978; **21(**4): 317-322.

4. **Azzalini A.** The R package 'sn': The Skew-Normal and Skew-t distributions (version 1.5-0). 2017.

5. **Coenen S, et al.** Clinical impact of five large-scale screening projects for chronic hepatitis B in Chinese migrants in the Netherlands. *Liver International* 2016; **36**(10): 1425-1432.

6. **GGD Amsterdam.** [Report with descriptive analyses China on the Amstel]. GGD Amsterdam, Department of Infectious Diseases; 2012.

7. **Niessen W, et al.** [Is inviting migrants for hepatitis B screening by letter post effective?]. *Infectieziekten Bulletin*. 2013; **24**(4): 107-111.

8. **Veldhuijzen IK, et al.** Viral hepatitis in a multi-ethnic neighborhood in the Netherlands: results of a community-based study in a low prevalence country. *International Journal of Infectious Diseases* 2009; **13**(1): e9-e13.

9. **Grintjes K, et al.** [Hepatitis C-detection among migrants in Nijmegen (HECOM)]. *Tijdschrift voor Infectieziekten.* 2014; **9**(5): 126-133.

10. **Zuure FR, et al.** Screening for hepatitis B and C in first-generation Egyptian migrants living in the Netherlands. *Liver International* 2013; **33**(5): 727-738.

11. **Baaten GG, et al.** Population-based study on the seroprevalence of hepatitis A, B, and C virus infection in Amsterdam, 2004. *J Med Virol* 2007; **79**(12): 1802-1810.

12. **Zuure FR, et al.** Prevalence of serological markers of hepatitis B virus and hepatitis C virus infection among six ethnic groups living in Amsterdam, the Netherlands – The HELIUS study. Poster presented at: *Global Viral Hepatitis Summit – 15th International Symposium on Viral Hepatitis and Liver Disease (ISVHLD)*; Berlin, Germany; 26-28 June 2015.

13. **Richter C, et al.** Screening for chronic hepatitis B and C in migrants from Afghanistan, Iran, Iraq, the former Soviet Republics, and Vietnam in the Arnhem region, The Netherlands. *Epidemiology & Infection* 2014; **142**(10): 2140-2146.

14. **Menger H, Neve G.** ['Vietnam among the tulips. Screening for hepatitis B for the Vietnamese population in North-Holland']. *Infectieziekten Bulletin.* 2013; **24**(2): 45-47.

15. **Bouwman-Notenboom AJ, et al.** ['China under the Dom: hepatitis B- and C-screening of Chinese migrants in Utrecht']. GGD Utrecht; 2011.

16. **hepatitisinfo.nl.** [Screening and awareness raising hepatitis B and hepatitis C among Chinese people in the Netherlands (2009-2013)] [cited 2017 June 12]. Available from: http://www.hepatitisinfo.nl/projecten/screening/DU2558_Screening-en-bewustzijnsbevordering-hepatitis-B-en-hepatitis-C-bij-Chinezen-in-Nederland-2009-2013.aspx.

17. **Urbanus AT, et al.** Hepatitis C in the general population of various ethnic origins living in the Netherlands: should non-Western migrants be screened? *Journal of Hepatology* 2011; **55**(6): 1207-1214.

18. **Kowdley KV, et al.** Prevalence of chronic hepatitis B among foreign-born persons living in the United States by country of origin. *Hepatology* 2012; **56**(2): 422-433.

19. **Gower E, et al.** Global epidemiology and genotype distribution of the hepatitis C virus infection. *Journal of Hepatology* 2014; **61**(1 Suppl): S45-57.
